# Educational Policies of Martial Art and Health in Schools Implemented by Japanese Colonial Power (1931–1937)

**Published:** 2018-07

**Authors:** Eui-Ryong HWANG, Tae-Young KIM

**Affiliations:** College of Education, Hankuk University of Foreign Studies, Seoul, Republic of Korea

## Dear Editor-in-Chief

Since the reformation of “Meiji restoration”, Japan transitioned to a new era with policies intended to integrate its nationals under imperialistic regulation. For this purpose, emphases were put on medicine and health including physical education (1). After annexation of Joseon, the Japanese colonial power promulgated “Educational decree of Joseon” in August of 1911. All educational activities in colony Joseon had to be based on it. The colonial government of Joseon additionally promulgated “Regulations for physical examination of students in government and public schools” in April of 1913 as an official order No. 24 pertaining to health in schools. Based on the decree, students participated in annual regular physical examination comprised of examinations for height, weight, chest girth, vertebral column, physique, visual acuity, presence of eye disease, auditory acuity, presence of ear disease, and teeth every April. Besides, the examination checked the presence of the following: malnutrition, anemia, glandular disorder, beriberi, pulmonary tuberculosis, headache, nervous prostration, throat disorder, infectious dermatoses, and other chronic diseases (2).

However, no study has explored realities of health, hygiene and martial art education implemented in schools of colony Joseon after the transition of Japanese colonial government to wartime formation with the momentum of Manchurian incident in 1931.

Therefore, the objective of this study was to examine the developmental process of practicing gymnastics in school and society which was employed to promote awareness of health of students in schools and determine the intention of Japanese colonial power reflected therein.

Changes in details taught in physical education practices in schools as well as policies of health implemented in schools were explored. With the outbreak of “Manchurian incident” in September of 1931, the bureau of educational affairs in the colonial government searched for directional change of school education to prepare for the upcoming war by identifying problems related to methods and contents of health education in schools (3). The bureau promulgated new educational directives in 1932. Contents ineligible for contemporary international situation found in “Necessary curricula for teaching gymnastics in school” were deleted (4). In particular, emphasis of health and hygiene education including physical education was put on the preparation of foundation in schools and extension of the education to families and society to maximize effects and satisfy contemporary needs that required close connection between families and society. The curriculum of health and hygiene employed in the department of gymnastics was also adopted by the department of science. Kyuba Fujii, a teacher in an elementary school, said the following, “In these days, firstly, the physique of female children in elementary schools gradually declines, thus, teachers should teach their students the physical structure of human body consists of skeleton, muscles, and skin etc. Secondly, structure and system of digestive organs should be taught. Thirdly, blood circulation, its function, and properties should be taught. Fourthly, functioning of respiratory organs and hygienic relationship thereto should be taught. Fifthly, the excretion and sanitation should be taught. Sixthly, the nervous system and hygienic relationship thereto should be taught. Seventhly, teachers should learn the anabolism and respiration of plants and teach students what they learned”, insisted that teachers should teach their gradually weakening students the human body by learning diverse components of human body correctly (5). However, contemporary schools in colony Joseon under the reign of Japanese colonial government were not provided with the environment to carry out educational decrees promulgated by the Japanese colonial government.

In June of 1936, the Japanese colonial government revised “Syllabus of gymnastics in schools” in Japan as they thought that the war would be intensified. Accordingly, the “Syllabus of Gymnastics in Schools” in Joseon was also revised by an official order No. 36 issued on May 29 of 1937 by the Japanese colonial government (6).

In particular, martial arts and military exercises in schools were emphasized in the perspective of cultivating military soldiers. Students in or over middle schools participated in the education of swords-manship, judo (male students), archery, Naginata, swimming, skiing, and skating (for all students). The revision of martial arts syllabus was oriented to intensify education of body and soul of students so that they can cope with forthcoming war. Thus, the curricula of physical education (swordsmanship, judo, swimming, and skating) enabled students to protect themselves from rugged natural environment. Essentials for fighting in a war were also introduced.

Along with the expansion of the war to regions in East Asia, the Japanese colonial power revised the “Syllabus of Gymnastics in Schools” wherein military exercises, swordsmanship, and judo were included as compulsory courses to let students to strengthen and improve their physical strength and physique so that they could adapt themselves to a war environment.

Wars in frigid area of Manchurian territory or under sultry weather of South-East Asia might have been presumed in advance. This was reflected in educational objectives of “Militarization of physical education” and “strengthening of physical force for the survival in severe natural environment” implemented in colony Joseon.
